# Needs Assessment for Research Use of High-Throughput Sequencing at a Large Academic Medical Center

**DOI:** 10.1371/journal.pone.0131166

**Published:** 2015-06-26

**Authors:** Albert Geskin, Elizabeth Legowski, Anish Chakka, Uma R Chandran, M. Michael Barmada, William A. LaFramboise, Jeremy Berg, Rebecca S. Jacobson

**Affiliations:** 1 Department of Biomedical Informatics, University of Pittsburgh School of Medicine, Pittsburgh, Pennsylvania, United States of America; 2 University of Pittsburgh Cancer Institute, Pittsburgh, Pennsylvania, United States of America; 3 Institute for Personalized Medicine, University of Pittsburgh School of Medicine, Pittsburgh, Pennsylvania, United States of America; 4 Department of Human Genetics, University of Pittsburgh Graduate School of Public Health, Pittsburgh, Pennsylvania, United States of America; CNRS UMR7622 & University Paris 6 Pierre-et-Marie-Curie, FRANCE

## Abstract

Next Generation Sequencing (NGS) methods are driving profound changes in biomedical research, with a growing impact on patient care. Many academic medical centers are evaluating potential models to prepare for the rapid increase in NGS information needs. This study sought to investigate (1) how and where sequencing data is generated and analyzed, (2) research objectives and goals for NGS, (3) workforce capacity and unmet needs, (4) storage capacity and unmet needs, (5) available and anticipated funding resources, and (6) future challenges. As a precursor to informed decision making at our institution, we undertook a systematic needs assessment of investigators using survey methods. We recruited 331 investigators from over 60 departments and divisions at the University of Pittsburgh Schools of Health Sciences and had 140 respondents, or a 42% response rate. Results suggest that both sequencing and analysis bottlenecks currently exist. Significant educational needs were identified, including both investigator-focused needs, such as selection of NGS methods suitable for specific research objectives, and program-focused needs, such as support for training an analytic workforce. The absence of centralized infrastructure was identified as an important institutional gap. Key principles for organizations managing this change were formulated based on the survey responses. This needs assessment provides an in-depth case study which may be useful to other academic medical centers as they identify and plan for future needs.

## Introduction

Next generation sequencing (NGS), with its high-throughput and range of applications, is revolutionizing life science research. NGS techniques are being used in research as diverse as the study of the microbiome [1], complexity of alternative splicing [2], and the mutational landscape in cancer [3]. NGS technologies are also beginning to impact the practice of medicine through the use of disease-targeted clinical sequencing [4], for example in cancer treatment selection [5, 6]. With further research, a more personalized, precise, and predictive model for medicine may be possible [7]. However, wider adoption of high throughput technology could also significantly stress academic medical centers, which are already burdened by decreased resources. To better understand what institutional resources are most important to support research communities that are adopting these technologies, we developed and administered an NGS needs assessment survey to a large number of biomedical researchers at a single university associated with an academic medical center (AMC).

## Background

Next generation sequencing techniques developed rapidly after the publication of the first draft of the human genome, with the introduction of massively parallel sequencing (MPS) technology in 2005 [8]. Parallelization of NGS technologies provided a faster, higher-throughput and lower cost method for sequencing than the traditional Sanger dideoxynucleotide sequencing method [9]. The cost of generating a whole human genome sequence reflected these advances: the cost has plummeted from the estimated price of $2.7 billion for the first genome draft sequence in 2001 to commercial sequencing costs of approximately $1,000 dollars for an entire genome in 2014 [10].

There are now a number of competing NGS platforms, including Complete Genomics, Illumina HiSeq, Life Technologies SOLiD and Ion Torrent, Pacific Biosciences SMRT, and Roche 454 [8, 11]. These platforms differ in sequencing chemistry, PCR amplification methods, read lengths, types of errors, overall error rates, and methods for downstream bioinformatics analysis [12]. Examples of specialized applications include whole genome sequencing (WGS) [13], whole exome sequencing (WES) [14], RNA-Seq [15], ChIP-Seq [16], chromatin conformation (Hi-C) [17], and Methyl-Seq [18], each with its own sophisticated data analysis pipeline. For researchers unfamiliar with these myriad technologies and applications, choosing the appropriate platform for a particular research question may appear to be a daunting challenge. The choice of platform is often determined by cost, institutional resources for sequencing, data management, storage, and bioinformatics capabilities. Our survey questions were designed to understand researchers’ levels of familiarity with these emerging technologies. Additionally, the questions sought to determine researchers’ current use of institutional versus external resources for each step of the sequencing pipeline.

Regardless of platform or application, NGS experiments generate large amounts of data–so called ‘Big Data’ [19, 20]–posing both data management and data analysis challenges. A single human whole genome sequence can generate 100–250 GB of data; therefore, even small research projects can quickly exceed the data storage capacity of individual labs [21]. It can be challenging for researchers to evaluate and implement the wide variety of storage solutions, each with various benefits and drawbacks. Cloud based storage and analysis solutions offer the benefits of no startup fees, relatively inexpensive long term storage, elasticity, and relatively rapid data access [22–25]. In comparison, keeping data locally in hard drives or storage arrays requires a one-time cost, provides vastly quicker data access, offers more direct control over security risks, and may have fewer regulatory complexities when compared to cloud based approaches [26]. From a budgetary perspective, all of these storage solutions are significantly more expensive than anything previously encountered in life science research. The NGS survey questions were designed to discern researchers’ familiarity with storage issues, including budgetary considerations, in order to guide future recommendations for institution-wide NGS infrastructure in networking and storage.

Data is considered the fourth paradigm for science, the first three being experimental, theoretical, and computational science [27]. NGS data offers unprecedented detail; however, the current rate of NGS data generation outpaces the rate at which we are able to analyze it. The complexity of NGS data analysis requires specialized interdisciplinary skills in biology, computing, information technology, and statistics [28, 29]. For example, a typical RNA Seq experiment analysis pipeline (alignment of reads to the reference genome, transcript quantification, and differential expression [30]) requires all of the above-mentioned skills. With the predicted increase in demand for data analysts and the lack of personnel with bioinformatics skills, NGS data analysis could become a significant bottleneck in biomedical research. Through this study, we also sought to understand the scope of the data analysis challenge, including researchers’ familiarity with computing resources such as cluster computing and whether individual investigators either have bioinformatics personnel in their laboratories or alternative solutions to meet their analysis needs.

## Materials and Methods

### Setting

University of Pittsburgh Health Sciences (UPHS) encompasses the Schools of Medicine, Public Health, Nursing, Dental medicine, Health and Rehabilitation, and Pharmacy, and is closely affiliated with UPMC, the single largest health care provider in western Pennsylvania. More than 2,000 individual researchers are included among the health science faculty. University of Pittsburgh is currently ranked number 5 in NIH funding. The Institute of Personalized Medicine was established in 2013 by University of Pittsburgh and UPMC to apply new knowledge in genetics, genomics, and other fields to advance evidence-based medicine.

### Survey Instrument

We developed an online survey instrument to elicit current and anticipated needs from researchers using Next Generation Sequencing (NGS) methods and data. Initial survey questions identified investigators who were currently using, planning on using, or not planning to use high throughput sequencing methods and to analyze resulting data. Investigators then responded to a set of survey questions specific to each of these three groups. Questions sought to elicit the following information: (1) how and where sequencing data is generated and analyzed, (2) research objectives and goals for NGS, (3) workforce capacity and unmet needs, (4) storage capacity and unmet needs, (5) available and anticipated funding resources, and (6) future challenges. Question types included multiple choice, Likert scale, ranking, rating scale, and fill-in-the-blank responses. Each participant answered a varying number of questions based on prior responses, ranging from a minimum of 9 and a maximum of 52 questions. The survey was created and administered on Survey Monkey.

### Recruitment

Participants were identified using searches performed on NIH RePORTER, PubMed, University of Pittsburgh Digital Vita research profiles, and University of Pittsburgh Schools of Health Sciences departmental websites. RePORTER and PubMed searches were specific to University of Pittsburgh and included one query specific to next generation sequencing, and another more broadly termed for genetic data. Institutional profile searches included terms relevant to sequencing and next generation sequencing. We also included faculty from departmental websites with stated interests in human genetics, human genomics, or sequencing. The final list was vetted by university scientific leaders to identify any investigators working in this area who were not already included. For each unique participant, we collected contact information, as well as School and Department or Division from the university directory.

### Participants

We invited participation of 331 investigators from over 60 departments and divisions at the University of Pittsburgh Schools of Health Sciences and at the Pittsburgh Supercomputing Center. Investigators were recruited by email invitation from institutional leaders containing a link to the survey. After the initial email, one subsequent reminder was mailed. A total of 140 respondents participated in the survey, a response rate of 42%. The research was approved as an Exempt study by the University of Pittsburgh IRB (PRO12110213). No consent was obtained. An informational script was used to (1) explain the purpose of the study, (2) describe how the data would be anonymized and protected, and (3) indicate that participants were free to withdraw at any time. The University of Pittsburgh IRB approved this procedure.

## Results

Results are grouped by whether researchers are currently using NGS, will use NGS in the future, or are not using or planning to use NGS data, depending on answers to initial survey questions (Table 1). The *All Users* group, representing 79% of the respondents, includes the 55% of investigators who are currently using NGS data (*Current Users*) as well as the 24% who are planning to use NGS data in the next 2 years (*Future Users*). The *Non Users* group, representing the other 21% of respondents, includes investigators who are neither currently using these methods, nor planning to use them in the next 2 years. For each group, the total number of respondents to any question may vary based on responses to prior questions. The reader is referred to supplemental material for a full listing of survey questions **(S1 File)** and for raw data **(S2 File)**.

**Table 1:** Number of respondents by use of NGS.

Label	Description	n (%)
**All Users**	Currently using NGS data OR plan to in next 2 years	**111/140 (79%)**
*Current Users*	*Currently using NGS data*	*77/140 (55%)*
*Future Users*	*Not currently using NGS data but plan to in next 2 years*	*34/140 (24%)*
**Non Users**	Not currently using NGS data and don’t plan to in next 2 years	**29/140 (21%)**

### How and where NGS data are being generated

Among *Current Users*, 43% are currently performing high throughput sequencing within the institution. Most of these respondents indicated that they used a core facility at their institution (58%) or a university collaborator’s lab (45%). Only 13% of researchers who are sequencing used an NG sequencer within their own laboratory. Among the 57% of *Current Users* who are not performing high throughput sequencing in the institution, 52% expect to be sequencing samples at the institution during the next two years. Eighty-two percent of these respondents expect to use a core facility at the university. In comparison, 77% of *Future Users* expect to begin sequencing within the institution during the next two years, and 94% of these respondents expect to use a core facility.

More than half of *Current Users* (56%) are outsourcing sequencing of samples to an external, non-university facility. Among these investigators, 45% are also sequencing within the university, while 55% are not performing any high-throughput sequencing within the university. Sixty percent of these respondents have sent samples to another academic institution for sequencing, and 43% have sent samples to a commercial sequencing service. Thirty-one percent of *Current Users* who are not yet outsourcing samples for sequencing expect to begin outsourcing in the next two years, with 50% of respondents planning to utilize academic institutions and 50% expecting to use commercial services.

### Where and how NGS data are being analyzed

Although the majority of the respondents did not sequence data at their laboratories, 61% of *Current Users* are analyzing NGS data within their own laboratory. Among investigators analyzing NGS data within their own lab, 45% are using both primary sequence and processed data, while 31% of respondents only use primary sequence data and 24% only use processed data. Additionally, 71% of the *Current Users* are running an NGS analysis pipeline in their own laboratory. Among investigators not analyzing NGS data within their own laboratory, 52% plan to send data to a collaborator for analysis. However, 50% of *Future Users* do not have any identified plans for analysis of NGS data.

Forty-six percent of *Current Users* are analyzing data from publicly available NGS datasets, with NCI’s The Cancer Genome Atlas (TCGA) being the most common dataset used (Table 2). Respondents reported that they used 13 different public NGS datasets, and 19% reported that they used multiple public datasets.

**Table 2:** Publicly available NGS datasets used for analysis (*Current Users*).

Dataset	n (%)
TCGA	11/32 (34%)
NCBI	2/32 (6%)
SRA	2/32 (6%)
1000 Genomes	2/32 (6%)
Numerous	6/32 (19%)
Other	9/32 (28%)
Not specified	1/32 (3%)

Investigators in the *All Users* group have samples ready to sequence that they have been unable to sequence, including 33% of *Current Users* and 24% of *Future Users* (Table 3). The most commonly cited obstacle to sequencing samples is cost, which 65% of respondents identified as a challenge. The second greatest obstacle, identified by 23% of respondents, is limited institutional resources. Additionally, across *All Users*, 31% of investigators have NGS sequencing data ready to analyze, but have been unable to do so. Among all the reasons investigators cited, lack of expertise and time are the main obstacles reported that have prevented them from analyzing data.

**Table 3:** Reasons respondents are unable to sequence samples and analyze sequences (*All Users*).

Category	Reason Cited by Respondent	n (%)
**Reason unable to sequence samples**	Cost/limited funds	20/31 (65%)
	Resources at university	7/31 (23%)
	Time/waiting for results	2/31 (6%)
	Other	2/31 (6%)
**Reason unable to analyze sequences**	Lack of Expertise	8/28 (29%)
	Time	6/28 (21%)
	Lack of help/support	3/28 (11%)
	Lack of resources	3/28 (11%)
	Funding	2/28 (7%)
	Too much data	2/28 (7%)
	Not complete dataset/recent acquisition of data	2/28 (7%)
	Low throughput by collaborator	1/28 (4%)
	Ongoing	1/28 (4%)

### Research objectives and applications/platforms

Researchers use NGS to meet a wide variety of research objectives (Table 4). From a domain perspective, 37% of *All Users* had cancer-related disease specific research objectives, and 39% had non-cancer specific disease research objectives. Less common objectives included using NGS for population biology, evolutionary biology, and metagenomics. From a task perspective, analysis of gene expression was the most frequently cited objective (54%), followed by systems modeling and prediction (22%), and discovery of novel transcripts (21%). Fewer than 20% of respondents had other objectives, such as protein-DNA binding (17%), small RNA discovery (17%), discovery of novel splice forms (13%), and DNA modification (10%).

**Table 4:** Research objectives of survey respondents (*All Users*).

Research Objectives	n (%)
Cancer disease-specific variants, structural variation, or copy-number changes	38/104 (37%)
Non-cancer disease-specific variants, structural variation, or copy-number changes	41/104 (39%)
Population biology	8/104 (8%)
Evolutionary biology	8/104 (8%)
Metagenomics	6/104 (6%)
DNA modification	10/104 (10%)
Protein-DNA binding	18/104 (17%)
Discovery of novel transcripts (gene discovery)	22/104 (21%)
Discovery of novel splice forms	13/104 (13%)
Small RNA discovery	18/104 (17%)
Gene expression	56/104 (54%)
Systems modeling and prediction	23/104 (22%)
Other	17/104 (16%)

Sixty-five percent of *All Users* cited RNAseq for gene expression as the application that best suited their research objectives, followed by targeted sequencing (41%), whole genome sequencing (39%), and whole exome sequencing (37%) (Table 5). Fewer than 10% of investigators are not sure what applications would best suit their objectives. Investigators most frequently identified sequencing by synthesis (Illumina: HiSeq or MiSeq) as the platform or method best suited to their objectives. However, many are not educated about the plethora of options. Despite their experience with NGS, 34% of *Current Users* are not sure what platform or method would be best. Of those users, most have not investigated platform options and do not know who to consult about platform options. *Future Users* are even less certain about platforms and methods, with 70% not sure what best suits their objectives.

**Table 5:** Applications and platforms/methods identified as best to suit objectives (*All Users*).

Category	Applications and Platforms/Methods	n (%)
**Applications identified to best suit objectives**	Targeted sequencing (Ampli-Seq or Target Seq)	43/104 (41%)
	Whole exome sequencing	38/104 (37%)
	Whole genome sequencing	41/104 (39%)
	RNAseq for gene expression	68/104 (65%)
	RNAseq for intron splice junctions (novel RNA discovery)	14/104 (13%)
	RNAseq for miRNA	28/104 (27%)
	MethylSeq	22/104 (21%)
	CHiPSeq	28/104 (27%)
	Not sure	9/104 (9%)
	Other	8/104 (8%)
**Platforms/methods identified to best suit objectives**	Ion semiconductor (Ion Torrent sequencing	29/104 (28%)
	Pyrosequencing (Roche 454)	13/104 (13%)
	Sequencing by synthesis (Illumina: HiSeq or MiSeq)	49/104 (47%)
	Sequencing by ligation (Life SOLiD sequencing)	7/104 (7%)
	Chain termination (Sanger sequencing)	11/104 (11%)
	Not sure	47/104 (45%)
	Other	8/104 (8%)

### Workforce: current capacity and unmet needs

Among investigators who analyze NGS data within their laboratory, the number of lab personnel primarily tasked with analyzing NGS data ranged from 0 to 6. Thirty-seven percent of these respondents report that they have a single individual primarily tasked with analyzing NGS data. The vast majority of researchers reported having fewer than four individuals tasked with NGS analysis, with 27% of investigators employing 2 and another 27% employing 3 lab personnel. Despite the existence of a nascent workforce, only 48% of respondents who are running an NGS analysis pipeline have any staff specifically trained in bioinformatics. These trained staff members have varying levels of education in bioinformatics (Table 6). Furthermore, 40% of respondents with trained staff indicate that some or all of their staff are entirely self-taught.

**Table 6:** Characteristics of Next Generation Sequencing workforce.

Category	Training and Skills	n (%)
***Staff members of Current Users trained in bioinformatics*: Level of education in bioinformatics**	Entirely self-taught	8/20 (40%)
	Bioinformatics short course	7/20 (35%)
	Masters in bioinformatics, computational biology, computer science, or a related field	8/20 (40%)
	PhD in bioinformatics, computational biology, computer science, or a related field	10/20 (50%)
**Skills one or more laboratory workers possess (*Current Users*)**	Unix and shell scripting	24/42 (57%)
	Object oriented programming	15/42 (36%)
	Database development and management	15/42 (36%)
	Statistical programming	22/42 (52%)
	Not sure	13/42 (31%)
**Skills sought in future NGS staff (*All Users*)**	Unix and shell scripting	15/23 (65%)
	Object oriented programming	16/23 (70%)
	Database development and management	13/23 (57%)
	Statistical programming	17/23 (74%)
	Other (genetic and medical models)	1/23 (4%)

Respondents reported that staff members had varied skills, but statistical programming and Unix and shell scripting were identified more frequently than object oriented programming and database development and management (Table 6). Across *All Users*, 26% expect to hire new staff to assist with future NGS analysis needs. A variety of skills will be sought in new staff, including statistical programming (74%), object oriented programming (70%), Unix and shell scripting (65%), and database development and management (57%).

### Storage methods: current capacity and unmet needs

We asked *Current Users* to assess their current data storage needs. Thirty-four percent of *Current Users* do not have the data storage capacity to handle their current NGS data needs. In addition, we asked *All Users* about future data storage needs. Only 10% felt they have the data storage capacity to handle their future needs. Over three-quarters (76%) of respondents expect to acquire additional storage in order to meet future storage demands. Among investigators who do not have the storage capacity to handle their current needs, 80% expect to acquire additional storage. However, there is no predominant storage method investigators expect to use, and 26% of respondents are not sure how they will meet their future storage needs.

Table 7 illustrates current and future data storage methods. The most common storage system was external hard drives, which 62% of respondents used. Many users also reported storing data on servers, either in their own laboratory (40%) or in other facilities (42%). Only 9% of respondents are currently using cloud storage. In the future, respondents expect to use external hard drives (82%) and servers within their labs (60%) at high levels. The largest projected increase is in the use of cloud storage, which 34% of *Current Users* expect to use in the future.

**Table 7:** Current and future storage methods for NGS data (*Current Users*).

Category	Storage Method	n (%)
**Currently use**	External hard drive	40/65 (62%)
	Servers (total)	44/65 (68%)
	Servers in lab	26/65 (40%)
	Servers outside lab	27/65 (42%)
	Cloud storage	6/65 (9%)
**Expect to use in future**	External hard drive	53/65 (82%)
	Servers (total)	47/64 (73%)
	Servers in lab	39/65 (60%)
	Servers outside lab	23/64 (36%)
	Cloud storage	22/65 (34%)

### Current and anticipated costs and funding for NGS sequencing and analysis

Most *Current Users* reported that they have not allocated large amounts of funding to NGS sequencing and analysis, but many respondents expected to increase their funding allocations in the future (Table 8). Among *Current Users*, 22% had allotted no funding for performing sequencing in the past 3 years, while only 10% allotted $100,000 or more per year. Forty-one percent of investigators allotted between $10,000 and $50,000 per year during this time. In comparison, analysis and storage of data were more commonly funded than sequencing. A greater number of researchers apportioned funds for analyzing and storing the data, with only 18% of respondents reporting that they had allotted no funding for the past 3 years. However, funding amounts for analyzing and storing data were much lower, with 44% of investigators allocating less than $10,000 per year.

**Table 8:** Funding allotted per year for Next Generation Sequencing (*Current Users*).

Category	Funding	n (%) Past 3 Years	n (%) Next 3 Years
**Funding allotted per year for performing sequencing**	None	15/68 (22%)	6/68 (9%)
	Less than $10,000	11/68 (16%)	6/68 (9%)
	$10,000-$49,999	28/68 (41%)	35/68 (51%)
	$50,000-$99,999	7/68 (10%)	13/68 (19%)
	$100,000-$250,000	5/68 (7%)	6/68 (9%)
	More than $250,000	2/68 (3%)	2/68 (3%)
**Funding allotted per year for analyzing and storing data**	None	12/68 (18%)	5/68 (7%)
	Less than $10,000	30/68 (44%)	22/68 (32%)
	$10,000-$49,999	16/68 (24%)	22/38 (32%)
	$50,000-$99,999	5/68 (7%)	7/68 (10%)
	$100,000-$250,000	3/68 (4%)	10/68 (15%)
	More than $250,000	2/68 (3%)	2/68 (3%)

Respondents plan to allot more funding in the future, with 82% expecting to allocate over $10,000 per year for sequencing over the next 3 years, compared to 62% who allotted over $10,000 per year in the past 3 years (Table 8). Additionally, a greater proportion of NGS users plan to allocate some funding in the future, with 91% of respondents planning to allot funding for performing sequencing and 93% planning to allot funding for analysis and data storage over the next 3 years.

### Challenges to NGS sequencing and analysis

We asked *All Users* about perceived challenges to analyzing and storing sequencing data, workflow, and cloud computing/storage (Table 9). Each item was rated on a scale from 1 (not at all challenging) to 5 (very challenging). All challenges were rated as somewhat difficult to overcome on average. Respondents (*n* = 77) perceived that sample preparation or library construction was the easiest task, with an average difficulty of 2.4. The two most challenging tasks were finding a person to perform the analysis (3.6) and how to meet the cost (3.7). Almost all items in the analyzing and storing sequencing data category were rated above average difficulty (3). This indicates that there are hurdles to overcome in all categories, but that they are not insurmountable.

**Table 9:** Challenges to use of Next Generation Sequencing (*All Users*).

Category	Challenges	Average Difficulty
**Challenges to analyzing and storing sequencing data**	Cost	3.7
	Finding a person to perform the analysis	3.6
	Access to computing power to perform the analysis	3.4
	Rapidly changing tools	3.3
	Management of the data	3.2
	Availability of storage space	3.2
	Lack of standardization of data formats	3.1
	Data transfer (networking)	3.1
	Difficulty of using open source software	3.1
	Compliance with regulations and policies	2.8
	Access control/security	2.7
	Other	1.0
**Challenges to workflow**	Data analysis and construction	3.5
	Moving the data along the workflow	3.1
	Storage	3.0
	Sharing the data with collaborators	2.7
	Sequencing	2.6
	Sample prep or library construction	2.4
**Challenges to use of cloud computing/storage**	Data transfer issues	3.4
	Cost	3.3
	Security	3.2
	Knowledge	3.2
	Availability	3.0
	Not advanced enough	2.9
	Other	2.3

### Knowledge among investigators who are not planning to use NGS

A total of 29 respondents are neither currently using nor planning to use NGS data (*Non Users*). Among these investigators, 38% have research questions that next generation sequencing can answer. Among respondents who have research questions that NGS can answer, 30% of respondents have investigated options for sequencing. Only 10% of these respondents know what technologies, methods, or platforms to use, but 78% report that they know where to find help to make decisions about which methods, technologies, or platforms to use. However, no respondents have investigated options for analysis or know what analysis software to use.

## Discussion

Advances in high throughput sequencing are fundamentally changing biomedical research and patient care–supporting a new paradigm of *personalized medicine* that includes genomic analysis as part of diagnostic and therapeutic decision-making [5, 6]. Although it provides significant promise, the ‘path to personalized medicine’ [7] is also likely to produce significant changes in the needs of the research community. Despite this emerging challenge, few roadmaps exist to help academic medical centers anticipate and plan for changing needs in this field. Following the establishment of the Institute for Personalized Medicine (IPM) at University of Pittsburgh, we undertook a systematic analysis of the data management needs of health sciences investigators who are either using or planning to use NGS methods. As a leading research institution (currently ranked 5^th^ in NIH funding) associated with a large vertically-integrated health care system (UPMC), this needs assessment provides an in-depth case study which may be of use to other AMCs as they identify and plan for future needs.

This discussion is organized around a set of key principles emerging from our survey, primarily focused on development of appropriate IT infrastructure, support of analytical resources, and education. Using these principles, our institution has launched specific initiatives designed to enhance our maturing ability to support the use of next generation sequencing for translational sciences. For each principle, we provide examples from our efforts, as well as limitations and pitfalls for development within this rapidly changing environment.

### 1. Cultivate strategic partnerships with research computing groups within the organization

The scale of NGS presents data management challenges not previously encountered by many institutions [31–33]. As AMCs develop information architectures, centers of expertise, and human processes to support personalized medicine initiatives, they can anticipate further strain on core resources [21]. Institutional support for data management will likely require partnerships that extend beyond traditional AMC boundaries, such as partnerships with industry and scientific computing centers. Forging such partnerships may be an important early step in program development.

An important early step at our institution was the development of a campus-wide strategic task force designed to specifically address the institutional aspects of managing this transition. In an effort to directly address the infrastructure needs elicited from participants, the Task Force and the Institute for Personalized Medicine assisted the Schools of the Health Sciences at University of Pittsburgh in developing strategic partnerships, both internally and externally. The Pittsburgh Supercomputing Center (PSC) [34] now hosts more than one petabyte of Pitt/UPMC NGS data on PSC’s proprietary Data Exacell parallel file system [35]–a set of high performance software and hardware building blocks for scientific computing, which is funded by the National Science Foundation. At the same time, we have significantly invested in the University Simulation and Modeling Center (SaM)[36], a centralized research computing group supporting several large-scale computing clusters. SaM provides high-quality, investigator-focused software and hardware resources, as well as consultants to assist researchers in moving their analysis pipelines to a clustered computing environment. These strategic partnerships have provided researchers using NGS methods at our University with access to significant storage and computational resources.

### 2. Build for high-throughput as well as high-performance computing needs

Results from our survey suggested a wide array of research objectives that require diverse computational infrastructures. High Performance Computing (HPC) environments provide parallel computing with infrastructures built for capability over capacity. In these types of environments, jobs typically require many hundreds or even thousands of CPUs, and potentially many gigabytes or even terabytes of memory, but very little storage. Job components need to communicate with one another over the entire set of CPUs. Under these conditions, large shared memory, message-passing interfaces, and low latency interconnects are essential for some important NGS computational tasks such as denovo RNA and DNA assembly. However, for most NGS computational tasks, the processing capacity may outweigh capability. In these situations, High Throughput Computing (HTC) architectures are typically preferred, allowing distribution of many thousands of jobs with low CPU requirements, and little to no inter-process communication, but much larger storage requirements. Bioinformatics pipelines can often be efficiently deployed in such HTC environments. As we develop our partnerships with the Simulation Modeling Center (SaM) and the Pittsburgh Supercomputing Center, which use both HPC and HTC environments, we are refining our understanding of how to use each set of resources to its best advantage, and how to best direct researchers to the appropriate resources for their work.

### 3. Develop centralized NGS data management as well as analysis

Centralized computing resources such as PSC and SaM provide important physical infrastructure including compute nodes, storage, networking, security, and support for managing the regulatory compliance aspects needed for NGS research [37]. But actual management of NGS data requires an investment beyond such physical infrastructure, including support for data provenance, integration, processing, and analysis. Specific centralized data management efforts can increase efficiency and reduce barriers to entry for investigators who are starting to use NGS in their labs. An advantage of centralizing data management infrastructure is that a diverse array of technologies can be employed to tier data based on the frequency with which the data must be accessed. Centralized infrastructure can also stage data between locations in advance of the movement to specific locations as part of the analysis workflow.

As an example, our survey showed that a large number of investigators were either currently using or interested in using TCGA data [38]. Consequently, we developed a process to automatically download, version, store, and update TCGA data (including BAM files) at the Pittsburgh Supercomputing Center, and to use the PSC distributed file system to make these same files available at the Simulation and Modeling Center. The process enables resources from both centers to be used with TCGA data. The Pittsburgh Genome Resource Repository (PGRR) supports a multi-investigator collaborative effort to use multi-institutional datasets such as TCGA for NGS analysis and personalized medicine. Collocating such large NGS datasets with relevant tools and compute resources at two research computing units has greatly enhanced the availability and utility of this dataset to our research community.

### 4. Anticipate future use of cloud-computing, while recognizing its limitations

Cloud-based storage and analysis are increasingly popular for NGS data [39], because of the relative flexibility, scalability, and affordability [22, 40–42]. Genomics cloud computing providers, such as Globus Genomics, Google Genomics, and Amazon Web Services, offer services using a variety of models and pipelines. More specific cloud-based bioinformatics workflow platforms provide further capabilities [43, 44]. These resources offer significant advantages for some NGS analysis use cases [24], particularly for researchers who have a single set of samples to examine. In contrast, for projects which are constantly accruing participants, or for ongoing efforts such as those envisioned in a personalized medicine setting, long-term storage of large data sets and repeated re-analysis of data make in-house computing resources far more cost effective than cloud providers. For investigators working with dbGAP datasets, Data Use Terms previously restricted use of cloud providers for protected data. But recent shifts in NIH policy [45], aligned with early experiments in NIH cloud-based resources, are significantly changing the landscape. Investigators in our institution are already using cloud resources for their own NGS projects, and we are beginning to consider how to leverage cloud providers through capabilities such as “cloud-bursting” (offloading jobs from overloaded computing resources in our institution to cloud providers on an as-needed basis). At the same time, many AMCs remain concerned about the wholesale use of cloud computing providers for sequencing data. Although research NGS data does not technically meet the definition of Protected Health Information, it is nonetheless sensitive personal information, incurring risk for re-identification or misuse [46]. New HIPAA-compliant cloud resources should help to alleviate these concerns, but will also require more significant investment for their use.

### 5. Consider institution-wide sequencing capacity and plan for sustainable growth

As shown in this survey, sequencing is an important bottleneck for NGS data processing. In our institutions, investigators are meeting their sequencing needs through a wide range of methods, from core resources to commercial providers. Choices are often specific to the needs of individual projects. Researchers may require an array of other services, from sample preparation to analysis and storage, as well as expertise in interpretation. Access to sequencing resources is becoming increasingly routine, and even amenable to “comparison shopping” through marketplace applications such as GenoHub [47]. In many respects, the key barrier is now cost. With a tightened NIH budget, traditional sources of funding are greatly reduced. Pilot programs such as those offered locally through Cancer Center Support Grants and Clinical and Translational Sciences Awards can provide vital seed funding for investigators to accrue preliminary data for grant applications. However, a more cohesive and long term strategy is needed to develop a sustainable model for funding NGS data generation. This could include negotiating larger volume contracts at reduced price and/or enhanced service, or explicitly funneling low complexity projects to commodity resources. Institutions that address these problems early on may benefit as demand increases.

### 6. Develop core analytic groups with financial model for escalating needs

NGS analysis requires specialized expertise that blends biological, statistical, computational, and communication skills [48]. AMCs can anticipate the need for more analytic capabilities as demand increases. While researchers can be expected to contribute to the costs of these core analytic groups, it is impractical to expect that all operational costs can be absorbed by the projects themselves. Using a cost-sharing model where a portion of the support for core analytical groups is provided by the University and a portion is shouldered by the investigators, we have increased our analytic capabilities significantly. As investigators request analytic help from our Bioinformatics Core Service, we apportion analysis time and hire new analysts as workloads increase. Institutional resources help cover intervals with lighter workloads, and enable hiring of new analysts before they reach 100% salary coverage.

### 7. Invest in bioinformatics training at all levels

Significant educational needs were identified in this rapidly changing domain. Individuals currently tasked with analyzing NGS data in individual labs often have very limited training in bioinformatics. To meet these immediate needs, workshops and other short-term programming for laboratory staff engaged in NGS analysis can provide an immediate way to address educational gaps. At our institution, an intensive three week summer NGS workshop run by one of our authors (MB) provides hands-on training in NGS analysis using datasets, tools, and infrastructures available at our institution. Over 250 individuals have attended this workshop in the last three years. This program fills a critical niche for training staff, students, and postdocs who are working with NGS data. An advantage of a workshop run within the institution is that it fosters development of a community among analysts in the research labs, and between these individuals and the analysts in the bioinformatics core services. Institutions also need to further invest in NGS by addressing educational gaps with long-term strategies, such as developing, sustaining, and expanding formal bioinformatics undergraduate and graduate programs. As a result of this survey and current trends in genomics and bioinformatics, we are expanding several existing graduate training programs and are partnering with other departments such as Computer Science and Information Science to develop innovative new training programs. Looking forward, federal support for programs that seek to retrain qualified bioscience PhDs and postdoctoral trainees may also help establish a larger bioinformatics workforce.

## Conclusions

This study provides an in-depth analysis of the current and planned use of NGS data analysis for health sciences research at a single academic medical center. Our findings suggest significant institutional challenges that AMCs can address to enhance their capacity for growth in genomic medicine, including infrastructure issues, support for centralized analytic resources, and education. Key principles for change management in this rapidly evolving space are presented. Based on these principles, our institution has taken initial steps towards development of Big Data infrastructure. Early strategic planning by AMCs will help to prevent the development of individually-targeted solutions, which may produce considerable fractionation of resources and duplication of services. Development of a campus-wide task force to address infrastructure and analytic issues is recommended to ensure that all members of the community benefit from the proposed changes.

## Supporting Information

S1 File NGS Needs Assessment Survey Instrument. The file includes the entire survey instrument as an enumeration of questions, question types, and enumerated answers, along with description of branching logic and the total number of individuals who were asked each question based on the branching logic.
(DOCX)

S2 File Raw Data from Survey Results. The file includes all counts used to generate results reported, including statements and tables.
(DOCX)
