# Supplementary material for: Needs Assessment for Research Use of High-Throughput Sequencing at a Large Academic Medical Center
Source: PLoS One. 2015 Jun 26;10(6):e0131166. doi: 10.1371/journal.pone.0131166 (PMC4483235; doi:10.1371/journal.pone.0131166)
Supplement: S1 File — The file includes the entire survey instrument as an enumeration of questions, question types, and enumerated answers, along with description of branching logic and the total number of individuals who were asked each question based on the branching logic. (DOCX) [file pone.0131166.s001.docx]

**S1 File. NGS Needs Assessment Survey Instrument.**

**Personalized Medicine Informatics Needs Assessment Survey Questions**

**Questions to target correct respondent population:**

1. Are you currently using next generation sequencing (NGS) data in your research? (y/n) (**N=141***)*

*If yes to Question 1, go to Question 2. If no to Question 1, skip to Question 3.*

2. Does your current research include human NGS data? (y/n) (**N=76**)

3. Are you planning to use next generation sequencing (NGS) data in your research within the next two years? (y/n) (**N=63**)

*If yes to Question 3 – ask Questions 4-5.*

4. Are you planning to use primary sequence (raw data) within the next two years (y/n)? (**N=34**)

5. Will your future research include human NGS data? (y/n) (**N=34**)

NOTE: Respondents who answer No to Questions 1 and 3 get separate short survey.

**For respondents who answered No to Questions 1 and 3:**

6. Do you have research questions that next generation sequencing can answer? (y/n) (**N=29**)

*If no to Question 6, go to Thank You page at end of survey.*

*If yes to Question 6, go to Question 7.*

7. Why are you not planning to use next-generation sequencing? (checklist) (**N=10**)

- Cost
- Lack of resources
- Don’t know where to start
- Other (please specify) __________________________

8. Have you investigated options for sequencing? (y/n with text box) (**N=10**)

- No
- Yes (please explain) ______________________

9. Have you investigated options for analysis? (y/n with text box) (**N=10**)

- No
- Yes (please explain) ______________________

10. Do you know what NGS technologies, methods, or platforms to use if you were to use next generation sequencing? (y/n with text box) (**N=10**)

- No
- Yes (please specify what you would use) _______________________________

*If no to Question 10, go to Question 11.*

*If yes to Question 10, skip to Question 12.*

11. Do you know where you could find help at University of Pittsburgh to make decisions about which methods, technologies, or platforms to use? (y/n) (**N=9**)

12. Do you know what analysis software to use with your anticipated results if you were to use next generation sequencing? (y/n with text box) (**N=10**)

- No
- Yes (please specify what you would use) ______________________

*If no to Question 12, go to Question 13.*

*If yes to Question 12, skip to Question 14.*

13. Do you know where you could find help at University of Pittsburgh to make decisions about which analysis software to use? (y/n) (**N=10**)

14. Do you know which institutional resources are available to you to perform NGS sequencing and analysis? (y/n) (**N=10**)

SKIP TO QUESTION 75

75. Would you be interested in having your name included in a Personalized Medicine Directory for development of personalized medicine research and clinical practices at Pitt? Only your name and contact information will be made available. (y/n) (**N=10**)

*If yes to Question 75, go to question 76.*

*If no to Question 75, skip to question 77.*

76. Would you be interested in being contacted to participate in a brief interview about your sequencing data needs? (y/n) (**N=7**)

*If yes to Question 76, skip to question 78.*

*If no to Question 76, go to Thank You page at end of survey.*

77. Would you be interested in being contacted to participate in a brief interview about your sequencing data needs? (y/n) (**N=3**)

*If yes to Question 77, go to question 78.*

*If no to Question 77, go to Thank You page at end of survey.*

78. Please provide the following information (for directory or interview purposes only): (**N=7**)

Name ________________________________________

Department ___________________________________

Email address __________________________________

Phone number _________________________________

GO TO THANK YOU PAGE AT END OF SURVEY

**For respondents who answer Yes to Question 1 or 3:**

**Collect information on use of sequencing technology:**

15. Is your laboratory currently performing high throughput sequencing within the University of Pittsburgh? (y/n) (**N=110**)

*If yes to Question 15, ask Questions 16-18, then skip to Question 23.*

*If no to Question 15, ask Questions 19-20.*

16. Which of the following facilities have you used? (checklist) (**N=35**)

- Genomics and Proteomics Core Facility (Director: Paul Wood at Gold Building)
- Cancer Biomarker Facility (Director: Bill LaFramboise at Shadyside Hospital)
- NG Sequencer within my own laboratory
- NG Sequencer in collaborators’ lab at Pitt (provide name of collaborator)

17. Which of the following NGS APPLICATIONS are you currently using? (checklist) (**N=35**)

- Targeted Sequencing (Ampli-Seq or Target Seq)
  - - Whole exome sequencing
    - Whole genome sequencing
    - RNAseq for gene expression or splice variant characterization or novel RNA discovery
    - RNAseq for miRNA
    - MethylSeq
    - CHiPSeq
    - None
    - Other (specify)_______________________

18. Which of the following sequencing PLATFORMS/METHODS are you currently using? (checklist) (**N=35**)

- Ion semiconductor (Ion Torrent sequencing)
- Pyrosequencing (Roche 454)
- Sequencing by synthesis (Illumina: HiSeq or MiSeq)
- Sequencing by ligation (Life SOLiD sequencing)
- Chain termination (Sanger sequencing)
- None
- Other (specify) _____________________

19. If you are not sequencing within your lab, please select any of the options your research involves: (checklist) (**N=71**)

- I am involved in using NGS data generated by a collaborator at another institution
- I am involved in using NGS data generated by a collaborator at University of Pittsburgh
- None
- Other (please specify) _______________________

20. Do you expect to be performing high throughput sequencing at the University of Pittsburgh during the next two years? (y/n) (**N=72**)

*If yes to Question 20, ask Questions 21-22.*

*If no to Question 20, skip to Question 23.*

21. When do you expect to be performing high throughput sequencing? (select one) (**N=43**)

- Within the next year
- Within the next 3 years
- Within the next 5 years
- 5 or more years from now

22. Which of the following facilities do you expect to use? (checklist) (**N=33**)

- Genomics and Proteomics Core Facility (Director: Paul Wood at Gold Building)
- Cancer Biomarker Facility (Director: Bill LaFramboise at Shadyside Hospital)
- NG Sequencer within my own laboratory
- NG Sequencer in collaborators lab at Pitt (provide name of collaborator:____________)

23. Is your laboratory ‘outsourcing’ the next generation sequencing of samples to a facility outside of the University of Pittsburgh? (y/n) (**N=105**)

*If yes to Question 23, go to Question 24, then skip to Question 27.*

*If no to Question 23, go to Question 25.*

24. Where have you sent samples to be sequenced: (checklist) (**N=54**)

- Another academic institution through a collaborative agreement or grant
- A commercial sequencing service
- Other

For each option selected, please specify where you have sent samples: ______________________

25. Do you expect that your laboratory will be ‘outsourcing’ next generation sequencing of samples to a facility outside of the University of Pittsburgh in the next two years? (y/n) (**N=51**)

*If yes to Question 25, go to Question 26.*

*If no to Question 25, skip to Question 27.*

26. Where do you expect to send samples to be sequenced? (checklist) (**N=15**)

- Another academic institution through a collaborative agreement or grant
- A commercial sequencing service
- Other

For each option selected, please specify where you have sent samples: ______________________

27. Do you currently have samples ready to sequence that you have not been able to sequence? (y/n with text box) (**N=103**)

- No
- Yes (please explain why you haven’t been able to sequence samples:___________)

28. What are your research objectives? (checklist) (**N=104**)

- Cancer disease-specific variants or structural variation or copy-number changes
- Non-cancer disease-specific variants or structural variation or copy-number changes
- Population biology
- Metagenomics
- Evolutionary biology
- DNA modification
- Protein-DNA binding
- Discovery of novel transcripts (gene discovery)
- Discovery of novel splice forms
- Small RNA discovery
- Gene expression
- Systems modeling and prediction
- Other _________________________________

29. Which APPLICATION(S) do you think would BEST SUIT your objectives? (checklist) (**N=104**)

- Targeted Sequencing (Ampli-Seq or Target Seq)
  - - Whole exome sequencing
    - Whole genome sequencing
    - RNAseq for gene expression
    - RNAseq for intron splice junctions (novel RNA discovery)
    - RNAseq for miRNA
    - MethylSeq
    - CHiPSeq
    - Not sure
    - Other (specify)__________________________

*If select Not sure, ask Questions 30-32.*

*Otherwise, skip to Question 33.*

30. Have you investigated NGS application options? (y/n) (**N=8**)

*If yes to Question 30, go to Question 31.*

*If no to Question 30, skip to Question 32.*

31. What have you done to investigate which NGS application options would best suit your research objectives? (text box) ________________ (**N=1**)

32. Do you know who to talk to at Pitt for consultation about application options? (y/n with text box) (**N=8**)

- No
- Yes (please specify which department) _________________________

33. Which sequencing PLATFORMS/METHODS do you think would BEST SUIT your objectives? (checklist) (**N=104**)

- Ion semiconductor (Ion Torrent sequencing)
- Pyrosequencing (Roche 454)
- Sequencing by synthesis (Illumina: HiSeq or MiSeq)
- Sequencing by ligation (Life SOLiD sequencing)
- Chain termination (Sanger sequencing)
- Not sure
- Other (specify) _______________________

*If select Not sure, ask Questions 34-36.*

*Otherwise, skip to Question 37.*

34. Have you investigated sequencing platform options? (y/n) (**N=38**)

*If yes to Question 34, go to Question 35.*

*If no to Question 34, skip to Question 36.*

35. What have you done to investigate which of these platforms would best suit your objectives? (text box) ______________________ (**N=5**)

36. Do you know who to talk to at Pitt for consultation about platform options? (y/n with text box) (**N=34**)

- No
- Yes (please specify which department) _________________

**Resources and capacity**

37. Is your laboratory analyzing data from publically available NGS datasets? (y/n with text box) (**N=100**)

- No
- Yes (please specify which datasets) _________________________

38. Are you analyzing NGS data within your own laboratory? (y/n) (**N=101**)

*If yes to Question 38, ask Questions 39-44, then skip to Question 46.*

*If no to Question 38, go to Question 45.*

39. What kind of data are you using? (checklist) (**N=44**)

- Primary sequence (raw) data
- Processed data

40. Do you currently run NGS analysis pipeline in your laboratory (alignment, variant calling, annotation, etc)? (y/n) (**N=46**)

41. How many individuals in your lab are currently primarily tasked with analyzing NGS data (include post-docs, graduate students, technicians and programmers)? (provide number selection 1-30) (**N=44**)

42. Do you have any staff members who are trained in bioinformatics? (y/n) (**N=46**)

*If yes to Question 42, go to Question 43.*

*If no to Question 42, skip to Question 44.*

43. Please indicate the level(s) of training of your staff members trained in bioinformatics. (checklist) (**N=22**)

- Entirely self-taught
- Bioinformatics short course
- Masters in bioinformatics, computational biology, computer science or a related field
- PhD in bioinformatics, computational biology, computer science or a related field

44. Which of the following skills does one or more member of your laboratory team currently possess? (checklist) (**N=46**)

- Unix and shell scripting
- Object oriented programming
- Database development and management
- Statistical programming
- Not sure

45. How do you plan to get data analyzed? (checklist) (**N=52**)

- Send to collaborator to analyze
- Outsource analysis
- No plans to analyze right now
- Other

For each option selected, please elaborate: __________________

46. Do you expect to hire new staff to assist with future NGS analysis needs? (y/n) (**N=101**)

*If yes to Question 46, go to Question 47.*

*If no to Question 46, skip to Question 48.*

47. Which of the following skills will you be seeking for new staff assisting with future NGS analysis needs? (checklist) (**N=23**)

- Unix and shell scripting
- Object oriented programming
- Database development and management
- Statistical programming
- Other (please specify) _________________________

48. Approximately how much funding have you allotted on average per year across all grants for PERFORMING SEQUENCING in the PAST 3 years? (select one) (**N=99**)

- None
- Less than $10,000
- $10,000 - $49,999
- $50,000 - $99,999
- $100,000 - $250,000
- More than $250,000

49. Approximately how much funding have you allotted on average per year across all grants for ANALYZING AND STORING sequencing data in the PAST 3 years? (select one) (**N=99**)

- None
- Less than $10,000
- $10,000 - $49,999
- $50,000 - $99,999
- $100,000 - $250,000
- More than $250,000

50. Approximately how much funding do you expect to allot on average per year across all grants for PERFORMING SEQUENCING in the NEXT 3 years? (select one) (**N=99**)

- None
- Less than $10,000
- $10,000 - $49,999
- $50,000 - $99,999
- $100,000 - $250,000
- More than $250,000

51. Approximately how much funding do you expect to allot on average per year across all grants for ANALYZING AND STORING sequencing data in the NEXT 3 years? (select one) (**N=99**)

- None
- Less than $10,000
- $10,000 - $49,999
- $50,000 - $99,999
- $100,000 - $250,000
- More than $250,000

**Data Storage and Analysis Needs**

52. Do you CURRENTLY use EXTERNAL HARD DRIVES to store sequencing data? (y/n) (**N=94**)

53. Do you CURRENTLY store sequencing data on SERVERS IN YOUR LABORATORY? (y/n) (**N=94**)

54. Do you CURRENTLY store sequencing data on SERVERS OUTSIDE OF YOUR LABORATORY? (y/n with text box) (**N=93**)

- No
- Yes (please specify where) ______________________

55. Do you CURRENTLY use CLOUD STORAGE for storing sequencing data? (y/n with text box) (**N=93**)

- No
- Yes (please specify which cloud) _____________________

56. Do you expect to store sequencing data on EXTERNAL HARD DRIVES in the FUTURE? (y/n) (**N=94**)

57. Do you expect to store sequencing data on SERVERS IN YOUR LABORATORY in the FUTURE? (y/n) (**N=94**)

58. Do you expect to store sequencing data on SERVERS OUTSIDE OF YOUR LABORATORY in the FUTURE? (y/n with text box) (**N=92**)

- No
- Yes (please specify where) ___________________________

59. Do you expect to use CLOUD STORAGE for storing sequencing data in the FUTURE? (y/n with text box) (**N=93**)

- No
- Yes (please specify which cloud) _____________________

60. Do you have the data storage capacity to handle your CURRENT NGS data needs? (y/n) (**N=94**)

61. Do you have the data storage capacity to handle your FUTURE NGS data needs? (select one) (**N=94**)

- Yes
- No
- Not sure

*If Yes or Not Sure to Question 61, ask Question 62.*

*If No to Question 61, ask Question 63.*

62. Do you expect to acquire additional storage to handle current or future needs? (y/n) (**N=50**)

63. Do you expect to acquire additional storage to handle current or future needs? (y/n with text box) (**N=44**)

- Yes
- No (please explain why you will not be acquiring additional storage) ______

*If yes to Question 63, go to Question 64.*

*If no to Question 63, skip to Question 65.*

64. How do you expect to meet your future storage needs? (text box) (**N=23**)

1. Please estimate the number of whole GENOME samples you expect to need to store over the next two years. (enter whole number from 0- 99999999) (**N=68**)
2. Please estimate the number of whole EXOME samples you expect to need to store over the next two years. (enter whole number from 0- 99999999) (**N=65**)
3. Do you currently have NGS sequencing data ready to analyze that you have not been able to analyze? (y/n) (**N=93**)

*If yes to Question 67, ask Questions 68-69.*

*If no to Question 67, skip to question 70.*

1. Please describe why you have not been able to analyze these sequences. (text box) (**N=28**)
2. What aspects of your workflow are not met by existing tools? Please describe the extent to which you are writing your own analysis programs for these tasks.(text box) (**N=20**)

**Challenges**

1. Please indicate on a scale from 1 (not at all challenging) to 5 (very challenging) how challenging each factor below is for your current or future research to STORING AND ANALYZING sequencing data. (scale) (**N=77**)

- Data transfer (networking)
- Access control/security
- Access to computing power to perform the analysis
- Compliance with regulations and policies
- Management of the data
- Finding a person to perform the analysis
- Lack of standardization of data formats
- Cost
- Availability of storage space
- Difficulty of using open source software
- Rapidly changing tools
- Other (please specify) __________________

1. Please describe any ways in which the challenges above have limited your research. (text box) (**N=37**)
2. Please indicate on a scale from 1 (not at all challenging) to 5 (very challenging) indicating how challenging each process is in your WORKFLOW for your current or future research. (scale) (**N=74**)

- Sample prep or library construction
- Sequencing
- Data analysis and construction
- Storage
- Moving the data along the workflow
- Sharing the data with collaborators

1. Please indicate on a scale from 1 (not at all challenging) to 5 (very challenging) how challenging each factor below is for use of CLOUD COMPUTING/STORAGE is for your current or future research. (scale) (**N=70**)

- Cost
- Availability
- Knowledge
- Not advanced enough
- Data transfer issues
- Security
- Other (please specify) _______________

1. Are there any other comments you would like to add? (text box) (**N=21**)

75. Would you be interested in having your name included in a Personalized Medicine Directory for development of personalized medicine research and clinical practices at Pitt? Only your name and contact information will be made available. (y/n) (**N=87**)

*If yes to Question 75, go to question 76.*

*If no to Question 75, skip to question 77.*

76. Would you be interested in being contacted to participate in a brief interview about your sequencing data needs? (y/n) (**N=68**)

*If yes to Question 76, skip to question 78.*

*If no to Question 76, go to Thank You page at end of survey.*

77. Would you be interested in being contacted to participate in a brief interview about your sequencing data needs? (y/n) (**N=19**)

*If yes to Question 77, go to question 78.*

*If no to Question 77, go to Thank You page at end of survey.*

78. Please provide the following information (for directory or interview purposes only): (**N=72**)

Name _________________________

Department _____________________

Email address ___________________

Phone number __________________

THANK YOU PAGE

Thank you for taking our NGS needs assessment survey. We appreciate your input!
