# Supplementary material for: Needs Assessment for Research Use of High-Throughput Sequencing at a Large Academic Medical Center
Source: PLoS One. 2015 Jun 26;10(6):e0131166. doi: 10.1371/journal.pone.0131166 (PMC4483235; doi:10.1371/journal.pone.0131166)
Supplement: S2 File — The file includes all counts used to generate results reported, including statements and tables. (DOCX) [file pone.0131166.s002.docx]

**S2 File. Raw Data from Survey Results.**

NGS Needs Assessment Survey Results

**Screening Questions:**

| **Q # *** | **Question** | **Group Reporting** | **n **** | **No**  **n (%)** | **Yes**  **n (%)** |
| --- | --- | --- | --- | --- | --- |
| 1 | Currently using NGS data in your research? | All Users | 140 | 63 (45%) | 77 (55%) |
| 2 | Current research include human NGS? | Current Users | 76 | 13 (17.1%) | 63 (82.9%) |
| 3 | Planning to use NGS in next 2 years? | Future and Non Users | 63 | 29 (46%) | 34 (54%) |
| 4 | Planning to use primary sequence (raw) data in next 2 years? | Future Users | 34 | 4 (11.8%) | 30 (88.2%) |
| 5 | Future research include human NGS data? | Future Users | 34 | 7 (20.6%) | 27 (79.4%) |

* Q # = Survey question number.

** n = Total number of respondents who answered the question.

**Short Survey:**

| **Q #** | **Question** | **Group Reporting** | **n** | **No**  **n (%)** | **Yes**  **n (%)** |
| --- | --- | --- | --- | --- | --- |
| 6 | Research questions that NGS can answer? | Non Users | 29 | 18 (62.1%) | 11 (37.9%) |
| 7 | Why not planning to use NGS? | Non Users | 10 |  |  |
|  | cost |  | 4 |  |  |
|  | lack of resources |  | 5 |  |  |
|  | don't know where to start |  | 0 |  |  |
|  | other |  | 5 |  |  |
| 8 | Have you investigated options for sequencing? | Non Users | 10 | 7 (70%) | 3 (30%) |
| 9 | Have you investigated options for analysis? | Non Users | 10 | 10 (100%) | 0 (0%) |
| 10 | Do you know what technologies, methods, platforms to use? | Non Users | 10 | 9 (90%) | 1 (10%) |
| 11 | Do you know where to find help? | Non Users | 9 | 2 (22.2%) | 7 (77.8%) |
| 12 | Do you know what analysis software to use for NGS? | Non Users | 10 | 10 (100%) | 0 (0%) |
| 13 | Do you know where to find help? | Non Users | 10 | 2 (20%) | 8 (80%) |
| 14 | Do you know which institutional resources are available to you to perform NGS sequencing analysis? | Non Users | 10 | 5 (50%) | 5 (50%) |
| 75 | Would you like your name included in a Personalized Medicine Directory? | Non Users | 10 | 3 (30%) | 7 (70%) |
| 76/77 | Would you like to do a brief interview? | Non Users | 10 | 8 (80%) | 2 (20%) |

**Lab Performing High Throughput Sequencing:**

| **Q #** | **Question** | **Group Reporting** | **n** | **No**  **n (%)** | **Yes**  **n (%)** |
| --- | --- | --- | --- | --- | --- |
| 15 | Is your lab currently performing high throughput sequencing within Pitt? | Current Users | 76 | 43 (56.6%) | 33 (43.4%) |
| 16 | Which facilities have you used? | Current Users | 31 |  |  |
|  | Core facility at university |  | 18 |  |  |
|  | NG Sequencer within my own laboratory |  | 4 |  |  |
|  | NG Sequencer in collaborators' lab at university |  | 14 |  |  |
| 17 | Which NGS applications are you using? | Current Users | 31 |  |  |
|  | Targeted Sequencing (Ampli-Seq or Target Seq) |  | 14 |  |  |
|  | Whole exome sequencing |  | 13 |  |  |
|  | Whole genome sequencing |  | 14 |  |  |
|  | RNAseq for gene expression or splice variant characterization or novel RNA discovery |  | 16 |  |  |
|  | RNAseq for miRNA |  | 9 |  |  |
|  | MethylSeq |  | 6 |  |  |
|  | CHiPSeq |  | 4 |  |  |
|  | None |  | 0 |  |  |
|  | Other |  | 4 |  |  |
| 18 | Which platforms/methods are you using? | Current Users | 31 |  |  |
|  | Ion semiconductor (Ion Torrent sequencing) |  | 14 |  |  |
|  | Pyrosequencing (Roche 454) |  | 9 |  |  |
|  | Sequencing by synthesis (Illumina: HiSeq or MiSeq) |  | 20 |  |  |
|  | Sequencing by ligation (LifeSOLiD sequencing) |  | 9 |  |  |
|  | Chain termination (Sanger sequencing) |  | 8 |  |  |
|  | None |  | 1 |  |  |
|  | Other |  | 2 |  |  |

**Plans for Users not Performing Sequencing in Lab:**

| **Q #** | **Question** | **Current Users** | **Future Users** |
| --- | --- | --- | --- |
| 19 | If not sequencing in your lab, select what your research involves | 42 | 29 |
|  | using NGS data generated by a collaborator at another institution | 24 | 12 |
|  | using NGS data generated by a collaborator at Pitt | 12 | 2 |
|  | None | 2 | 12 |
|  | Other | 13 | 5 |
| 20 | Do you expect to be performing high throughput sequencing at Pitt during the next 2 years? | 42 | 30 |
|  | No | 20 | 7 |
|  | Yes | 22 | 23 |
| 21 | When do you expect to be performing high throughput sequencing? | 43 |  |
|  | Within the next year | 19 |  |
|  | Within the next 3 years | 20 |  |
|  | Within the next 5 years | 4 |  |
|  | 5 or more years from now | 0 |  |
| 22 | Which of the following facilities do you expect to use? | 17 | 16 |
|  | Core facility at university | 14 | 15 |
|  | NG Sequencer within my own laboratory | 0 | 0 |
|  | NG Sequencer in collaborators' lab at Pitt | 3 | 1 |

**Lab Outsourcing:**

| **Q #** | **Question** | **Group Reporting** | **n** | **No**  **n (%)** | **Yes**  **n (%)** |
| --- | --- | --- | --- | --- | --- |
| 23 | Is your lab outsourcing sequencing? | Current Users | 72 | 32 (44.%) | 40 (55.6%) |
| 24 | Where have you sent samples? | Current Users | 40 |  |  |
|  | Another academic institution |  | 24 |  |  |
|  | A commercial sequencing service |  | 17 |  |  |
|  | Other |  | 9 |  |  |
| 25 | Do you expect your lab will be outsourcing sequencing in the next 2 years? | Current Users | 32 | 22 (68.8%) | 10 (31.3%) |
| 26 | Where do you expect to send samples? | Current Users | 10 |  |  |
|  | Another academic institution |  | 5 |  |  |
|  | A commercial sequencing service |  | 5 |  |  |
|  | Other |  | 0 |  |  |
| 27 | Do you currently have samples ready to sequence that you have been unable to sequence? | All Users | 103 | 72 (69.9%) | 31 (30.1)% |
|  | Why not able to sequence? |  |  |  |  |
|  | cost/limited funds |  | 20 |  |  |
|  | resources at Pitt |  | 7 |  |  |
|  | time/waiting for results |  | 2 |  |  |
|  | other |  | 2 |  |  |

**Objectives:**

| **Q #** | **Question** | **Group Reporting** | **n** | **No**  **n (%)** | **Yes**  **n (%)** |
| --- | --- | --- | --- | --- | --- |
| 28 | What are your research objectives? | All Users | 104 |  |  |
|  | Cancer disease-specific variants or structural variation or copy-number changes |  | 38 |  |  |
|  | Non-cancer disease-specific variants or structural variation or copy-number changes |  | 41 |  |  |
|  | Population biology |  | 8 |  |  |
|  | Evolutionary biology |  | 8 |  |  |
|  | Metagenomics |  | 6 |  |  |
|  | DNA modification |  | 10 |  |  |
|  | Protein-DNA binding |  | 18 |  |  |
|  | Discovery of novel transcripts (gene discovery) |  | 22 |  |  |
|  | Discovery of novel splice forms |  | 13 |  |  |
|  | Small RNA discovery |  | 18 |  |  |
|  | Gene expression |  | 56 |  |  |
|  | Systems modeling and prediction |  | 23 |  |  |
|  | Other |  | 17 |  |  |
| 29 | Which applications do you think would best suit your objectives? | All Users | 104 |  |  |
|  | Targeted Sequencing (Ampli-Seq or Target Seq) |  | 43 |  |  |
|  | Whole exome sequencing |  | 38 |  |  |
|  | Whole genome sequencing |  | 41 |  |  |
|  | RNAseq for gene expression |  | 68 |  |  |
|  | RNAseq for intron splice junctions (novel RNA discovery) |  | 14 |  |  |
|  | RNAseq for miRNA |  | 28 |  |  |
|  | MethylSeq |  | 22 |  |  |
|  | CHiPSeq |  | 28 |  |  |
|  | Not sure |  | 9 |  |  |
|  | Other |  | 8 |  |  |
| 30 | Have you investigated NGS application options? | All Users | 8 | 7 (87.5%) | 1 (12.5%) |
| 31 | What have you done to investigate application options? | All Users | 1 |  |  |
|  | consultations |  | 1 |  |  |
| 32 | Do you know who to talk to at Pitt about application options? | All Users | 8 | 4 (50%) | 4 (50%) |
|  | Computational Biology |  | 1 |  |  |
|  | Pathology |  | 1 |  |  |
|  | HUGEN |  | 1 |  |  |
|  | GPCL? |  | 1 |  |  |

| **Q #** | **Question** | **Group Reporting** | **n** | **No**  **n (%)** | **Yes**  **n (%)** |
| --- | --- | --- | --- | --- | --- |
| 33 | Which platforms/methods do you think would best suit your objectives? | All Users | 104 |  |  |
|  | Ion semiconductor (Ion Torrent sequencing) |  | 29 |  |  |
|  | Pyrosequencing (Roche 454) |  | 13 |  |  |
|  | Sequencing by synthesis (Illumina: HiSeq or MiSeq) |  | 49 |  |  |
|  | Sequencing by ligation (Life SOLiD sequencing) |  | 7 |  |  |
|  | Chain termination (Sanger sequencing) |  | 11 |  |  |
|  | Not sure |  | 47 |  |  |
|  | Other |  | 8 |  |  |
| 34 | Have you investigated sequencing platform options? | All Users | 38 | 30 (79%) | 8 (21.1%) |
| 35 | What have you done to investigate which of these platforms best suits your objectives? | All Users | 5 |  |  |
|  | read articles and information about the different available platforms. Talked with colleagues. |  | 1 |  |  |
|  | consultations |  | 1 |  |  |
|  | Read papers. Talk with colleagues. Go to talks |  | 1 |  |  |
|  | Attended Illumina seminar |  | 1 |  |  |
|  | Talked with GPCL and research on internet |  | 1 |  |  |
| 36 | Do you know who to talk to at Pitt about platform options? | All Users | 34 | 21 (61.8%) | 13 (38.2%) |
|  | GPCL/cores |  | 6 |  |  |
|  | Human genetics |  | 3 |  |  |
|  | Pathology |  | 2 |  |  |
|  | DBMI |  | 1 |  |  |
|  | colleagues |  | 1 |  |  |

**Resources:**

| **Q #** | **Question** | **Group Reporting** | **n** | **No**  **n (%)** | **Yes**  **n (%)** |
| --- | --- | --- | --- | --- | --- |
| 37 | Is your laboratory analyzing data from publically available NGS datasets? | Current Users | 69 | 37 (53.6%) | 32 (46.4%) |
|  | TCGA |  | 11 |  |  |
|  | NCBI |  | 2 |  |  |
|  | SRA |  | 2 |  |  |
|  | 1000 Genomes |  | 2 |  |  |
|  | numerous |  | 6 |  |  |
|  | not specified |  | 1 |  |  |
|  | other |  | 9 |  |  |
|  | Genome Variation Server (U of Washington)  USCS  plasmodb.org  toxoplasma gondii gene expression and human host cell gene expression  blast N  df  ARRA Autism Sequencing Consortium, Simons Simplex Collection  Mouse Genomes by Stratin  Hapmap |  |  |  |  |
| 38 | Are you analyzing NGS data within your laboratory? | Current Users | 69 | 27 (39.1%) | 42 (60.9%) |
| 39 | What kind of data are you using? | Current Users | 42 |  |  |
|  | only primary sequence (raw) data |  | 13 |  |  |
|  | only processed data |  | 10 |  |  |
|  | both primary sequence and processed data |  | 19 |  |  |
| 40 | Do you currently run NGS analysis pipeline in your laboratory (alignment, variant calling, annotation, etc)? | Current Users | 42 | 12 (28.6%) | 30 (71.4%) |
| 41 | How many individuals in your lab are currently primarily tasked with analyzing NGS data (include post-docs, graduate students, technicians, and programmers)? | Current Users | 41 |  |  |
|  | Average |  | 1.98 |  |  |
|  | min |  | 0 |  |  |
|  | max |  | 6 |  |  |
| 42 | Do you have any staff members who are trained in bioinformatics? | Current Users | 42 | 22 (52.4%) | 20 (47.6%) |
| 43 | Please indicate the level(s) of training of your staff members trained in bioinformatics. | Current Users | 20 |  |  |
|  | Entirely self taught |  | 8 |  |  |
|  | Bioinformatics short course |  | 7 |  |  |
|  | Masters in bioinformatics, computational biology, computer science or a related field |  | 8 |  |  |
|  | PhD in bioinformatics, computational biology, computer science or a related field |  | 10 |  |  |
| **Q #** | **Question** | **Group Reporting** | **n** | **No**  **n (%)** | **Yes**  **n (%)** |
| 44 | Which of the following skills does one or more member of your laboratory team currently possess? | Current Users | 42 |  |  |
|  | Unix and shell scripting |  | 24 |  |  |
|  | Object oriented programming |  | 15 |  |  |
|  | Database development and management |  | 15 |  |  |
|  | Statistical programming |  | 22 |  |  |
|  | Not sure |  | 13 |  |  |
| 45 | How do you plan to get data analyzed? | All Users | 52 |  |  |
|  | Send to collaborator to analyze |  | 27 |  |  |
|  | Outsource analysis |  | 12 |  |  |
|  | No plans to analyze right now |  | 13 |  |  |
|  | Other |  | 8 |  |  |
|  | analyze myself |  | 3 |  |  |
|  | collaborators |  | 9 |  |  |
|  | cores at Pitt |  | 3 |  |  |
| 46 | Do you expect to hire new staff to assist with future NGS analysis needs? | All Users | 101 | 75 (74.3%) | 26 (25.7%) |
| 47 | Which of the following skills will you be seeking for new staff assisting with future NGS analysis needs? | All Users | 23 |  |  |
|  | Unix and shell scripting |  | 15 |  |  |
|  | Object oriented programming |  | 16 |  |  |
|  | Database development and management |  | 13 |  |  |
|  | Statistical programming |  | 17 |  |  |
|  | Other (Genetic and medical models) |  | 1 |  |  |

**Funding:**

| **Q #** | **Question** | **Group Reporting** | **n** | **None**  **n (%)** | **Less than $10,000**  **n (%)** | **$10,000-$49,999**  **n (%)** | **$50,000 - $99,999**  **n (%)** | **$100,000 - $250,000**  **n (%)** | **More than $250,000**  **n (%)** |
| --- | --- | --- | --- | --- | --- | --- | --- | --- | --- |
| 48 | Average funding allotted for performing sequencing per year for past 3 years | Current Users | 68 | 15 (22.1%) | 11 (16.2%) | 28 (41.2%) | 7 (10.3%) | 5 (7.4%) | 2 (2.9%) |
| 49 | Average funding allotted for analyzing and storing sequencing data per year for past 3 years | Current Users | 68 | 12 (17.7%) | 30 (44.1%) | 16 (23.5%) | 5 (7.4%) | 3 (4.4%) | 2 (2.9%) |
| 50 | Average funding alloted for performing sequencing per year in next 3 years | Current Users | 68 | 6 (8.8%) | 6 (8.8%) | 35 (51.5%) | 13 (19.1%) | 6 (8.8%) | 2 (2.9%) |
| 51 | Average funding allotted for analyzing and storing sequencing data per year in next 3 years | Current Users | 68 | 5 (7.4%) | 22 (32.4%) | 22 (32.4%) | 7 (10.3%) | 10 (14.7%) | 2 (2.9%) |

| **Q #** | **Question** | **Group Reporting** | **n** | **None**  **n (%)** | **Less than $10,000**  **n (%)** | **$10,000-$49,999**  **n (%)** | **$50,000 - $99,999**  **n (%)** | **$100,000 - $250,000**  **n (%)** | **More than $250,000**  **n (%)** |
| --- | --- | --- | --- | --- | --- | --- | --- | --- | --- |
| 50 | Average funding allotted for performing sequencing per year in next 3 years | Future Users | 31 | 7 (22.6%) | 6 (19.4%) | 11 (35.5%) | 2 (6.5%) | 4 (12.9%) | 1 (3.2%) |
| 51 | Average funding allotted for analyzing and storing sequencing data per year in next 3 years | Future Users | 31 | 9 (29%) | 7 (22.6%) | 10 (32.3%) | 3 (9.7%) | 1 (3.2%) | 1 (3.2%) |

**Storage:**

| **Q #** | **Question** | **Current Users** | | | **Future Users** | | |
| --- | --- | --- | --- | --- | --- | --- | --- |
|  |  | **n** | **No**  **n (%)** | **Yes**  **n (%)** | **n** | **No**  **n (%)** | **Yes**  **n (%)** |
| 52 | Currently store on external hard drives | 65 | 25 (38.5%) | 40 (61.5%) | 29 | 23 (79.3%) | 6 (20.7%) |
| 53 | Currently store on servers in your laboratory | 65 | 39 (60%) | 26 (40%) | 29 | 20 (69%) | 9 (31%) |
| 54 | Currently store on servers outside your laboratory | 65 | 38 (58.5%) | 27 (41.5%) | 28 | 28 (100%) | 0 (0%) |
| 55 | Currently store on cloud storage | 65 | 59 (90.8%) | 6 (9.2%) | 28 | 28 (100%) | 0 (0%) |
| 56 | Expect to store on external hard drives in future | 65 | 12 (18.5%) | 53 (81.5%) | 29 | 12 (41.4%) | 17 (58.6%) |
| 57 | Expect to store on servers in your laboratory in the future | 65 | 26 (40%) | 39 (60%) | 29 | 15 (51.7%) | 14 (48.3%) |
| 58 | Expect to store on servers outside your laboratory in the future | 64 | 41 (64.1%) | 23 (35.9%) | 28 | 21 (75%) | 7 (25%) |
| 59 | Expect to store on cloud storage in the future | 65 | 43 (66.2%) | 22 (33.9%) | 28 | 24 (85.7%) | 4 (14.3%) |
| 60 | Have data storage capacity to handle current NGS data needs | 65 | 22 (33.9%) | 43 (66.2%) | ---- | ---- | ---- |

**Future Storage Needs:**

| **Q #** | **Question** | **Group Reporting** | **n** | **No**  **n (%)** | **Yes**  **n (%)** | **Not Sure**  **n (%)** |
| --- | --- | --- | --- | --- | --- | --- |
| 61 | Have data storage capacity to handle future NGS data needs | All Users | 94 | 44 (46.8%) | 9 (9.6%) | 41 (43.6%) |
| 62 | Expect to acquire additional storage for current or future needs | All Users | 50 | 14 (28%) | 36 (72%) |  |
| 63 | Expect to acquire additional storage for current or future needs | All Users | 44 | 9 (20.5%) | 35 (79.6%) |  |
|  | why not acquiring additional storage |  |  |  |  |  |
|  | institutional solution |  | 1 |  |  |  |
|  | not planned by institution |  | 1 |  |  |  |
|  | not sure |  | 4 |  |  |  |
|  | stored elsewhere |  | 1 |  |  |  |
|  | has adequate storage |  | 1 |  |  |  |
|  | depend upon collaborator |  | 1 |  |  |  |
| 64 | How do you expect to meet your future storage needs? | All Users | 23 |  |  |  |
|  | external hard drives |  | 4 |  |  |  |
|  | Pitt resources |  | 3 |  |  |  |
|  | servers or cloud |  | 3 |  |  |  |
|  | PCS |  | 2 |  |  |  |
|  | CHP server |  | 1 |  |  |  |
|  | MWRI server |  | 1 |  |  |  |
|  | collaborating institution |  | 1 |  |  |  |
|  | purchase advanced storage options |  | 1 |  |  |  |
|  | purchase storage off site |  | 1 |  |  |  |
|  | not sure |  | 6 |  |  |  |
| 67 | Currently have NGS sequencing data ready to analyze that you haven't been able to analyze | All Users | 93 | 64 (68.8%) | 29 (31.2%) |  |
| 68 | Describe why you haven't been able to analyze | All Users | 28 |  |  |  |
|  | lack of expertise |  | 8 |  |  |  |
|  | time |  | 6 |  |  |  |
|  | lack of help/support |  | 3 |  |  |  |
|  | lack of resources |  | 3 |  |  |  |
|  | funding |  | 2 |  |  |  |
|  | too much data |  | 2 |  |  |  |
|  | not complete dataset/recent acquisition of data |  | 2 |  |  |  |
|  | low throughput by collaborator |  | 1 |  |  |  |
|  | ongoing |  | 1 |  |  |  |

**Number of Whole Genome and Exome Samples Expect to Store:**

| **Q #** | **Question** | **Group Reporting** | **n** | **Mean** | **Median** | **Min** | **Max** | **Total** |
| --- | --- | --- | --- | --- | --- | --- | --- | --- |
| 65 | Estimate number of whole genome samples you expect to need to store in next 2 years | All Users | 68 | 197 | 10 | 0 | 6,000 | 13,403 |
| 66 | Estimate number of whole exome samples you expect to need to store in next 2 years | All Users | 65 | 434 | 12 | 0 | 20,000 | 28,179 |

**Challenges** (scale of 1 to 5 with 1 meaning not at all challenging and 5 meaning very challenging):

| **Challenges to Analyzing and Storing Sequencing Data** | **Group Reporting** | **n** | **Average Rating** | **n/a** |
| --- | --- | --- | --- | --- |
| Data transfer (networking) | All Users | 77 | 3.07 | 5 |
| Access control/security | All Users | 77 | 2.74 | 5 |
| Access to computing power to perform the analysis | All Users | 77 | 3.42 | 3 |
| Compliance with regulations and policies | All Users | 75 | 2.83 | 6 |
| Management of the data | All Users | 77 | 3.23 | 2 |
| Finding a person to perform the analysis | All Users | 75 | 3.62 | 4 |
| Lack of standardization of data formats | All Users | 77 | 3.11 | 7 |
| Cost | All Users | 76 | 3.74 | 6 |
| Availability of storage space | All Users | 77 | 3.16 | 2 |
| Difficulty of using open source software | All Users | 77 | 3.14 | 7 |
| Rapidly changing tools | All Users | 75 | 3.29 | 6 |
| Other | All Users | 11 | 1 | 9 |

| **Challenges to Workflow** | **Group Reporting** | **n** | **Average Rating** | **n/a** |
| --- | --- | --- | --- | --- |
| Sample prep or library construction | All Users | 72 | 2.36 | 13 |
| Sequencing | All Users | 74 | 2.56 | 10 |
| Data analysis and construction | All Users | 74 | 3.52 | 5 |
| Storage | All Users | 74 | 3.04 | 5 |
| Moving the data along the workflow | All Users | 73 | 3.14 | 7 |
| Sharing the data with collaborators | All Users | 73 | 2.73 | 9 |

| **Challenges to use of cloud computing/storage** | **Group Reporting** | **n** | **Average Rating** | **n/a** |
| --- | --- | --- | --- | --- |
| Cost | All Users | 70 | 3.27 | 25 |
| Availability | All Users | 70 | 3.04 | 23 |
| Knowledge | All Users | 70 | 3.17 | 23 |
| Not advanced enough | All Users | 69 | 2.86 | 26 |
| Data transfer issues | All Users | 70 | 3.38 | 23 |
| Security | All Users | 70 | 3.2 | 24 |
| Other | All Users | 27 | 2.33 | 24 |

**Directory/Interview:**

| **Q #** | **Question** | **Group Reporting** | **n who answered** | **No** | **Yes** | **No (%)** | **Yes (%)** |
| --- | --- | --- | --- | --- | --- | --- | --- |
| 75 | Would you like your name included in a Personalized Medicine Directory? | All Respondents | 97 | 22 | 75 | 22.68 | 77.32 |
| 76/77 | Would you like to do a brief interview? | All Respondents | 97 | 43 | 54 | 44.33 | 55.67 |

| **Q #** | **Question** | **n who got question** | **n who answered** | **No** | **Yes** | **No (%)** | **Yes (%)** |
| --- | --- | --- | --- | --- | --- | --- | --- |
| 75 | Would you like your name included in a Personalized Medicine Directory? | All Users | 87 | 19 | 68 | 21.84 | 78.16 |
| 76/77 | Would you like to do a brief interview? | All Users | 87 | 35 | 52 | 40.23 | 59.77 |

**Comparison of Applications:**

| **Application** | **Currently Using** | **Suits Objectives** | **Same People** |
| --- | --- | --- | --- |
| Targeted Sequencing (Ampli-Seq or Target Seq) | 16 | 15 | 13 |
| Whole exome sequencing | 13 | 14 | 11 |
| Whole genome sequencing | 14 | 16 | 13 |
| RNAseq for gene expression or splice variant characterization or novel RNA discovery | 16 | 25 | 15 |
| RNAseq for miRNA | 9 | 12 | 7 |
| MethylSeq | 6 | 10 | 5 |
| CHiPSeq | 4 | 13 | 3 |
| None/NotSure | 2 | 2 | 1 |
| Other | 4 | 2 | 1 |

**Comparison of Platforms/Methods:**

| **Platform/Method** | **Currently Using** | **Suits Objectives** | **Same People** |
| --- | --- | --- | --- |
| Ion semiconductor (Ion Torrent sequencing) | 14 | 19 | 12 |
| Pyrosequencing (Roche 454) | 10 | 8 | 6 |
| Sequencing by synthesis (Illumina: HiSeq or MiSeq) | 20 | 23 | 18 |
| Sequencing by ligation (LifeSOLiD sequencing) | 9 | 5 | 2 |
| Chain termination (Sanger sequencing) | 10 | 6 | 6 |
| None/Not Sure | 3 | 8 | 2 |
| Other | 2 | 2 | 0 |
